# Description of the calf thymus DNA-malathion complex behavior by multi-spectroscopic and molecular modeling techniques: EMF at low and high frequency approaches

**DOI:** 10.22038/IJBMS.2021.58083.12907

**Published:** 2021-10

**Authors:** Tahmineh Sohrabi, Maryam Asadzadeh-Lotfabad, Zahra Shafie, Zeinab Amiri Tehranizadeh, Mohammad Reza Saberi, Jamshidkhan Chamani

**Affiliations:** 1 Department of Biology, Faculty of Sciences, Mashhad Branch, Islamic Azad University, Mashhad, Iran; 2 Medical Chemistry Department, School of Pharmacy, Mashhad University of Medical Sciences, Mashhad, Iran

**Keywords:** ctDNA, Electromagnetic field, Groove binding, Spectroscopy, Viscosity

## Abstract

**Objective(s):**

Small molecules can bind to DNA via covalent or non-covalent interactions, which results in altering or inhibiting the function of DNA. Thus, understanding the interaction patterns of medicines or other small molecules can be very crucial. In this study, the interaction between malathion and calf thymus DNA (ctDNA), in the absence and presence of electromagnetic field (EMF) at low and high frequencies, was investigated through various spectroscopies and viscosity measurements.

**Materials and Methods:**

The interaction studies were performed by means of absorbance, circular dichroism, fluorescence spectroscopy, viscosity, thermal melting, and molecular modeling techniques.

**Results:**

The fluorescence intensity of the ctDNA-malathion complex in the presence of EMF, has revealed quenching of fluorescence emission curves. The dynamic interaction and RLS studies have implied the changes in ctDNA-malathion complex throughout the presence of EMF which suggested that hydrophobic forces play the main role in the binding. Studies have revealed that malathion does not have any effect on binding ethidium bromide to ctDNA, which signifies the groove binding. The viscosity of ctDNA increased as the malathion concentration was enlarged. The circular dichroism technique suggested that the ellipticity values of the ctDNA-malathion complex have not increased with enhancing the malathion concentration. Molecular docking and dynamics studies have indicated a potent electrostatic interaction between ctDNA and malathion in the groove binding site.

**Conclusion:**

The results of spectroscopic studies reinforced a potent interaction between malathion and ctDNA in the absence and presence of EMF which can help us for further pharmaceutical drug discoveries.

## Introduction

Deoxyribonucleic acid (DNA) was discovered in the cell nucleus by Mischer in 1871 and for a long time, it did not attract much attention since a polymer that is composed of four simple bases was assumed to be only capable of functioning in supportive and not major roles, which was in contrast to the proteins that are arranged from 20 different amino acids (1). The common studies on DNA often focus on their interaction with molecules, such as small organic compounds and metal complexes, and the results usually assist in understanding the mechanism of such molecules with biopolymers while providing some guidance for designing new drugs as well (2). The interaction of small molecules, such as drugs (3-5) and organic dyes (6-8), with DNA, is a subject that stands at the interface of chemistry and biology. As it is known, the intercellular target for the majority of anticancer, antibiotic, and antiviral drugs is DNA (9). Small molecules can bind to DNA via covalent or non-covalent interactions, which results in altering or inhibiting the function of DNA (10, 11). There are several types of sites in the DNA molecules where the binding of ligand complexes can occur, including the two base pairs (intercalation), within the minor or major grooves, and on the outside of the helix. It is notable that intercalative binding is stronger than the other three binding modes since the intercalative molecule is sandwiched in between the aromatic and heterocyclic base pairs of DNA (12).

**Scheme 1:**
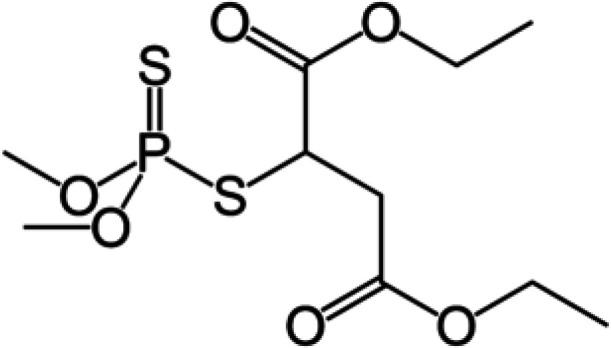
Malathion structure

Malathion (O,O-dimethyl-S-1,2-bis ethoxy carbonyl ethyl phosphorodithioate, Scheme 1) is an organophosphorus (OP) compound that is widely used in agriculture and veterinary practices as well as in attempts of suicide (13), eradicating ectoparasites and household insects, conserving stored grain, and eliminating disease-inducing arthropods(14-16). In addition, malathion is effective in controlling many insects such as leaf-eating caterpillars, thrips, cockchafer larvae, cutworms, etc., along with a range of certain crops that include vegetables, fruits, maize, sugar cane, sugar beet, tea, tobacco, and ornamentals (17). The OPs can infiltrate all the tissues and due to their lipophilic nature and simple and rapid intestinal assimilation eventually lead to several pathological difficulties that include insufficiency of the immune system (18, 19), pancreatitis (20), liver disease (21, 22), hematological pathosis disorder (23), and reduce the fertility and reproduction capability (24). Herein, we have studied the interaction between malathion and ctDNA, in the absence and presence of electromagnetic fields at low and high frequencies, resulting in discovering the affinity of this substance to ctDNA with different sides. The binding investigation of ctDNA and malathion as an OP compound that is widely used in agriculture and veterinary practices as well as in attempts of suicide and determination of the binding site of the malathion on the ctDNA can reveal the usage of malathion doses for agricultural and veterinary practices. The obtained outcomes can be utilized for decreasing the toxicity properties of malathion upon interaction with ctDNA.

## Materials and Methods

***Chemicals and reagents***

Malathion, ctDNA, EB, and AO were obtained from Sigma Chemical Co. (St. Louis, Mo. USA), and used without purification. We have procured Tris-HCl buffer from Merck Chemical Co. ctDNA was dissolved in 10 mM of Tris buffer solution pH 6.8. Then, the malathion solution (0.1 mM) was provided by being dissolved in Tris buffer. EB (3.2 × 10^-4^ M) and AO (2 × 10^-7^ M) were dissolved in 10 mM of Tris buffer solution at pH 6.8. The filtered ctDNA solution gave an ultraviolet (UV) absorbance ratio (A_260_/A_280_) of 1.9, indicating that ctDNA had been sufficiently freed from protein (25, 26). The molar extinction coefficient and concentration of ctDNA were observed to be 6600 M^-1^ cm^-1^ and 1.37 × 10^-4^ M, respectively (27, 28).

***Methods***

*Spectrophotometric measurements*

The required fluorescence measurements were performed at room temperature through the usage of a F-2500 spectrofluorometer (Hitachi, Japan). In the absence and presence of electromagnetic fields, fluorescence emission spectra were measured to be at 298, 303, and 308 K, which existed in the wavelength range of 280–300 nm with an excitation wavelength of 260 nm. The competitive interaction between malathion, EB, and AO as intercalator probes with ctDNA was carried out as follows: fixed amounts of EB, AO, and ctDNA were titrated by increasing the portions of the malathion solution. The available AO and EB were excited at 490 nm and 440 nm, respectively, while the emission were recorded between 500–700 nm for AO and 500–800 nm in the case of EB.

We performed the procedure of UV-visible absorption spectra with a Jasco V-630 spectrophotometer, while the solutions of ctDNA and malathion were scanned in a 1 cm quartz cuvette. This experiment was carried out in the absence and presence of electromagnetic fields. The UV spectra of ctDNA-malathion, in the absence and presence of electromagnetic fields, were detected to be from 200 nm to 800 nm. The effects of ionic strength on the interaction between malathion and ctDNA were investigated by varying the concentrations of NaCl and KI in the absence and presence of electromagnetic fields. 

The melting experiments of ctDNA were carried out by monitoring the absorption of ctDNA at 260 nm in the absence and presence of electromagnetic fields at various temperatures, which involved the utilization of a spectrophotometer that was coupled to a thermocouple. The melting transition (T_m_) of ctDNA-malathion was determined as a transition midpoint. We recorded the Circular dichroism (CD) measurements on a Jasco (J-815, Japan) spectropolarimeter at room temperature, in which the scan range was from 240 nm to 300 nm. A spectrum of Tris-HCl buffer at pH 6.8 was detected from the spectra of ctDNA-malathion in the absence and presence of EMF. The process of the inner filter effect was discharged for all experiments (29, 30).

*Viscosity measurements*

Viscosity measurements were carried out through the application of an Ostwald viscometer that had been thermostated at 298 K at a constant temperature. We have presented the data as (µ/µ_0_)^1/3^ of ctDNA solution versus the malathion concentration, where µ_0_ and µ represent the viscosities of ctDNA and ctDNA-malathion solutions in the absence and presence of electromagnetic fields, respectively. In similar conditions, the concentration of ctDNA-malathion complex in the Tris-HCl buffer solution (pH=6.8) was fixed while the flow time was measured by a digital stopwatch. 

*Molecular modeling*

The structure of a sample B-DNA molecule was obtained from Protein Data Bank (RCSB accession ID: 1BNA). The designated molecule (DNA (5’-D(*CP*GP*CP*GP*AP*AP*TP*TP*CP*GP*CP*G)-3’)) was optimized in case of structural defects and charges in Molecular Operating Environment (MOE®). In addition, the structure of malathion was drawn in ChemOffice software and saved in the MOL2 format for further optimization by MOE. We optimized both ctDNA and malathion through the following method prior to performing the docking procedure. Initially, they were protonated with the Protonate3D tool at 300 K temperature and pH 7. Then, the salts concentration was considered to be 0.1 M in an implicit water model. Thereafter, the electrostatic interactions were calculated by means of Generalized Born / Volume Integral formalism (GB/VI) between two atoms with a 10 Å cutoff. To finish the process, ligand (malathion) and macromolecule (B-DNA) were energy minimized by the usage of Amber forcefield. The docking procedure was performed in vacuum, which enabled the possibility of using the whole receptor atoms as possible active sites, while 100 docking poses were collected in each docking. We have screened the obtained results by utilization of the London dG scoring matrix. The first- and second-best docking results were introduced to GROMACS software for dynamic studies (31) and we employed SwissParam web server to build the ligand topology files (32). Ligand and DNA were placed in a TIP3P water model box using the available all-atom GROMACS forcefields (33). During the steepest descend minimization, NVT and NPT thermostat, position restraints were applied on both ligand and ctDNA. Finally, molecular dynamics (MD) simulations were performed for 100 ns and the number of hydrogen bonds (H bonds) and electrostatic potentials between ctDNA and malathion were calculated during the course of the procedure. We have also evaluated the stability of the final complex from the beginning to the end of the MD simulation.

## Results

***Resonance light scattering (RLS) measurements***

**Figure 1:**
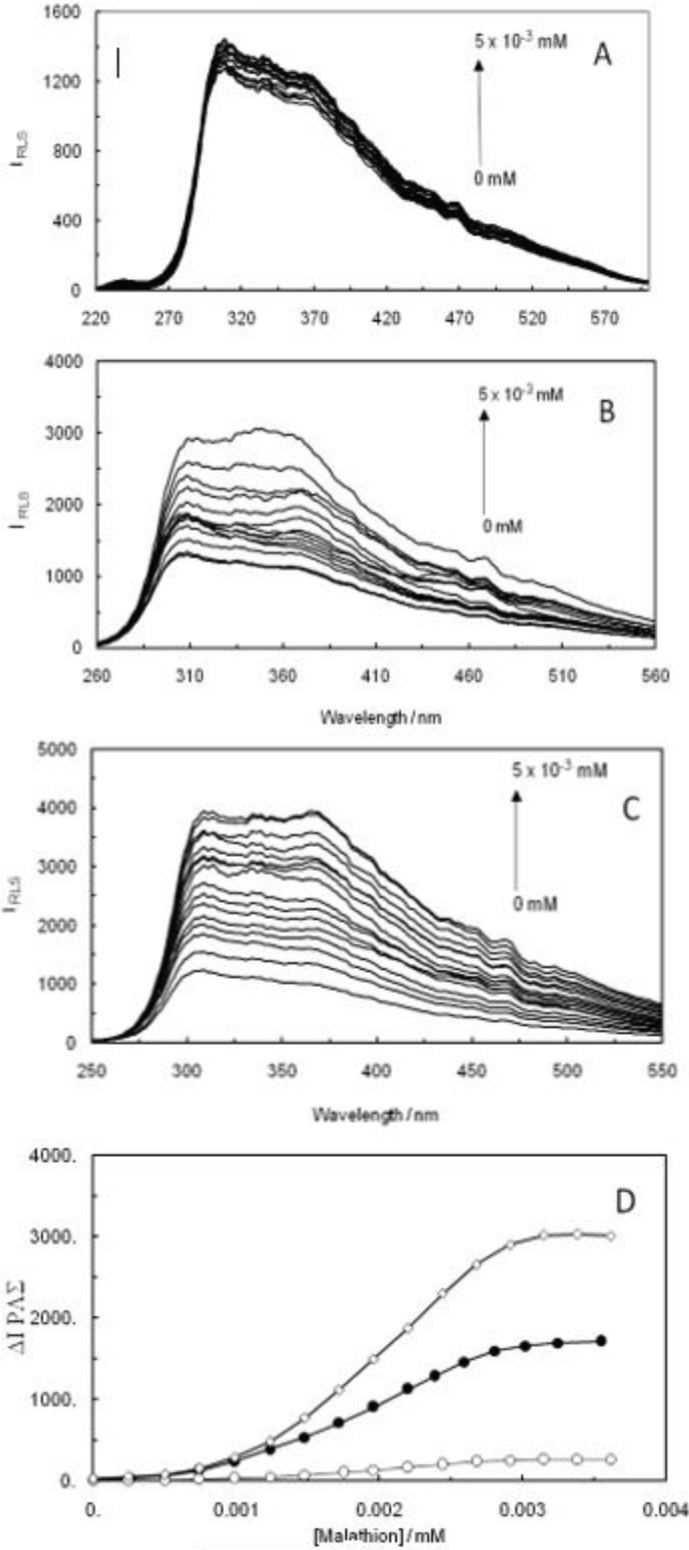
(A); RLS spectra of ctDNA-Malathion. (B); ctDNA-Malathion in the presence of 100 KHz electromagnetic field. (C); ctDNA-Malathion in the presence of 1.2 GHz electromagnetic field. (D); comparing ∆IRLS curve against the concentration of Malathion. ctDNA-Malathion (open circles); ctDNA-Malathion in the presence of 100 KHz electromagnetic field (closed circles); and ctDNA-Malathion in the presence of 1.2 GHz electromagnetic field (open diamonds)

The RLS results of ctDNA-malathion were obtained in the absence and presence of EMF (100 kHz, 1.2 GHz) (Figure 1). The regularly induced enhancement in RLS that had been caused by increasing the concentration of malathion to ctDNA indicated that an interaction had occurred between ctDNA-malathion in the absence and presence of EMF, which is shown in Figure 1(A-C). As shown in Figure 1, the RLS values of ctDNA-malathion in the presence of a 1.2 GHz electromagnetic field have differed from those of the ctDNA-malathion and ctDNA-malathion in the presence of 100 kHz EMF; on the other hand, an increase in the intensity of RLS can cause the binding of malathion to ctDNA in the absence and presence of EMF. Figure 1D demonstrates the curves of ∆IRLS versus the malathion concentration in regard to the ctDNA-malathion in the absence and presence of EMF (100 kHz, 1.2 GHz). According to this figure, the ∆IRLS values of ctDNA-malathion in the presence of 1.2 GHz EMF were higher than those of ctDNA-malathion in the presence of 100 kHz EMF. This fact suggests that the structural changes of ctDNA-malathion in the presence of 1.2 GHz EMF were different from ctDNA-malathion and ctDNA-malathion complexes in the presence of 100 kHz EMF. 

***Competitive interaction of AO and EB binding to ctDNA-malathion complex in the presence of EMF***

**Figure 2:**
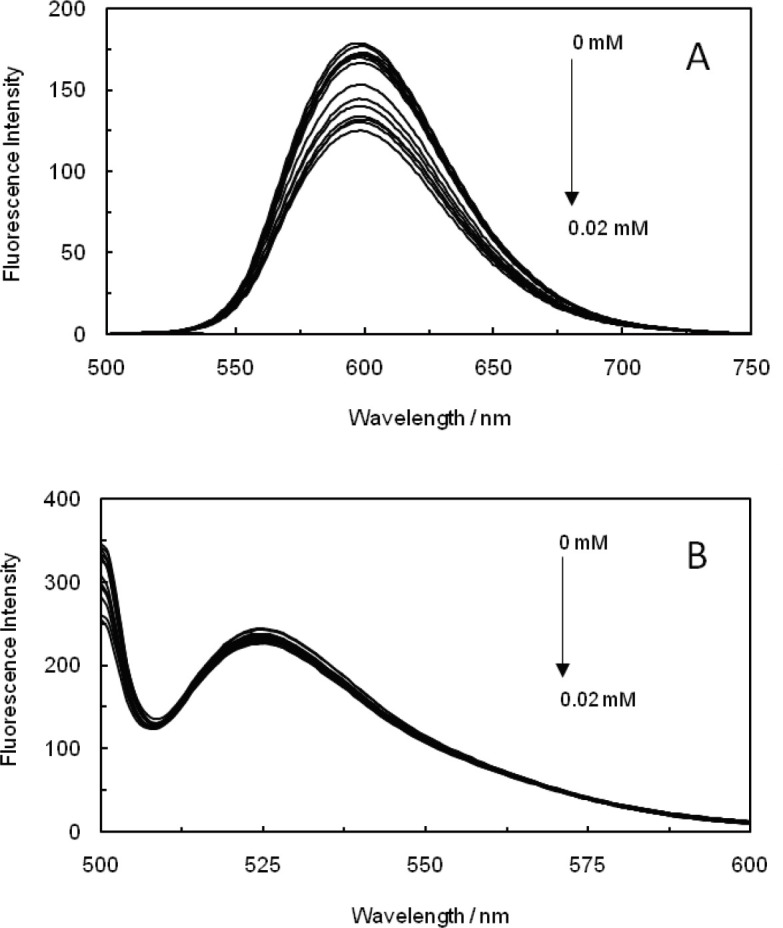
(A); Fluorescence spectra of the competition between Malathion and EB in the (ctDNA-EB-Malathion) system. (B); Fluorescence spectra of the competition between Malathion and AO in the (ctDNA-AO-Malathion) system

As it is known, the enhanced fluorescence of EB upon addition of ctDNA could be quenched, at least partly, through the appending of a second molecule (34). AO was widely used to study the binding mechanism that exists between molecules and DNA in various biological systems, which can intercalate between the two adjacent base pairs in DNA helix and increase the fluorescence intensity throughout fluorescence spectroscopy experiments (35). As Figure 2A demonstrates the fluorescence emission spectra of (EB-ctDNA) malathion, it is notable that there has not been any significant decrease in fluorescence intensity as the concentration of malathion was increased; thus, it is indicated that malathion binds to ctDNA through a non-intercalative mode. The emission spectra of (AO-ctDNA) malathion are displayed in Figure 2B, and as it can be observed, increasing the concentration of malathion has not caused any notable decrease in the fluorescence intensity of (AO-ctDNA) malathion, which proves the fact that malathion is not capable of replacing AO. This result once again has confirmed the fact that malathion does not bound to ctDNA through an intercalative mode. However, to confirm the probability of minor groove binding, another dye displacement experiment was performed by utilization of a known minor groove binder. The competitive interaction of AO and EB binding to ctDNA-malathion complex, which had been in the presence of EMF gave similar results in the absence of electromagnetic field (data is not illustrated). 

***Fluorescence measurements***

**Figure 3:**
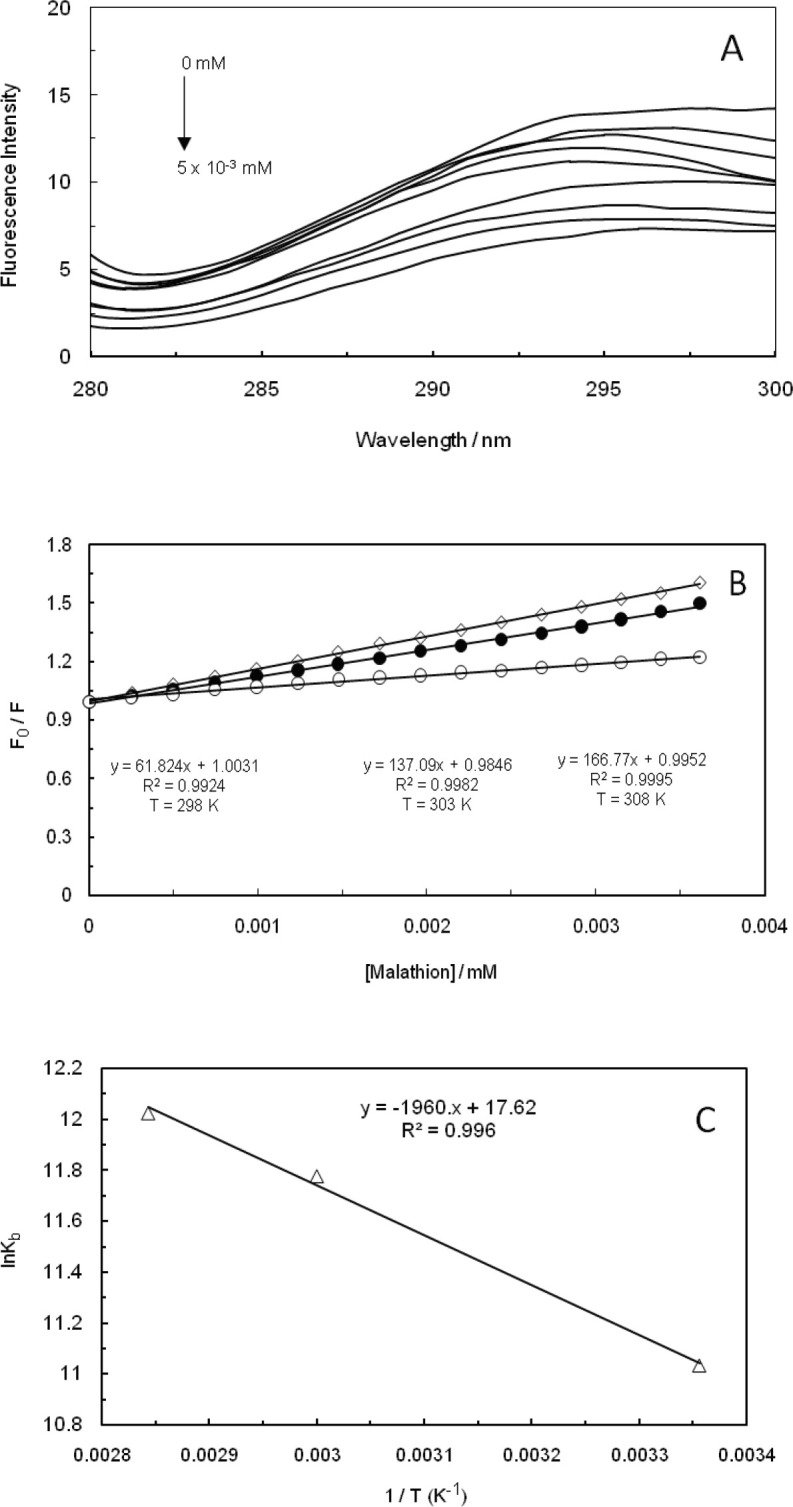
(A); Fluorescence spectra of ctDNA-Malathion at different concentration (λ_ex_= 260 nm). (B); Stern-Volmer plots of the fluorescence quenching of the ctDNA-Malathion at different temperatures. (C); Van't Hoff plot for the interaction of ctDNA-Malathion

**Figure 4:**
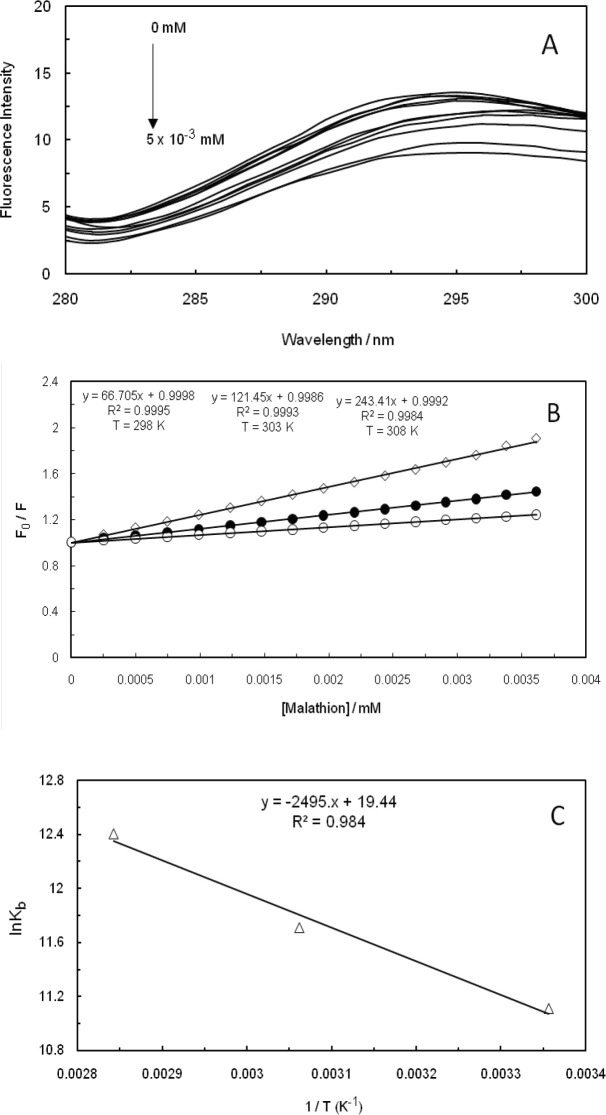
(A); Fluorescence spectra of ctDNA-Malathion in the presence of 100 KHz electromagnetic field at different concentration (λex= 260 nm). (B); Stern-Volmer plots of the fluorescence quenching of the ctDNA-Malathion in the presence of 100 KHz electromagnetic field at different temperatures. (C); Van't Hoff plot for the interaction of ctDNA-Malathion in the presence of 100 KHz electromagnetic field

**Figure 5:**
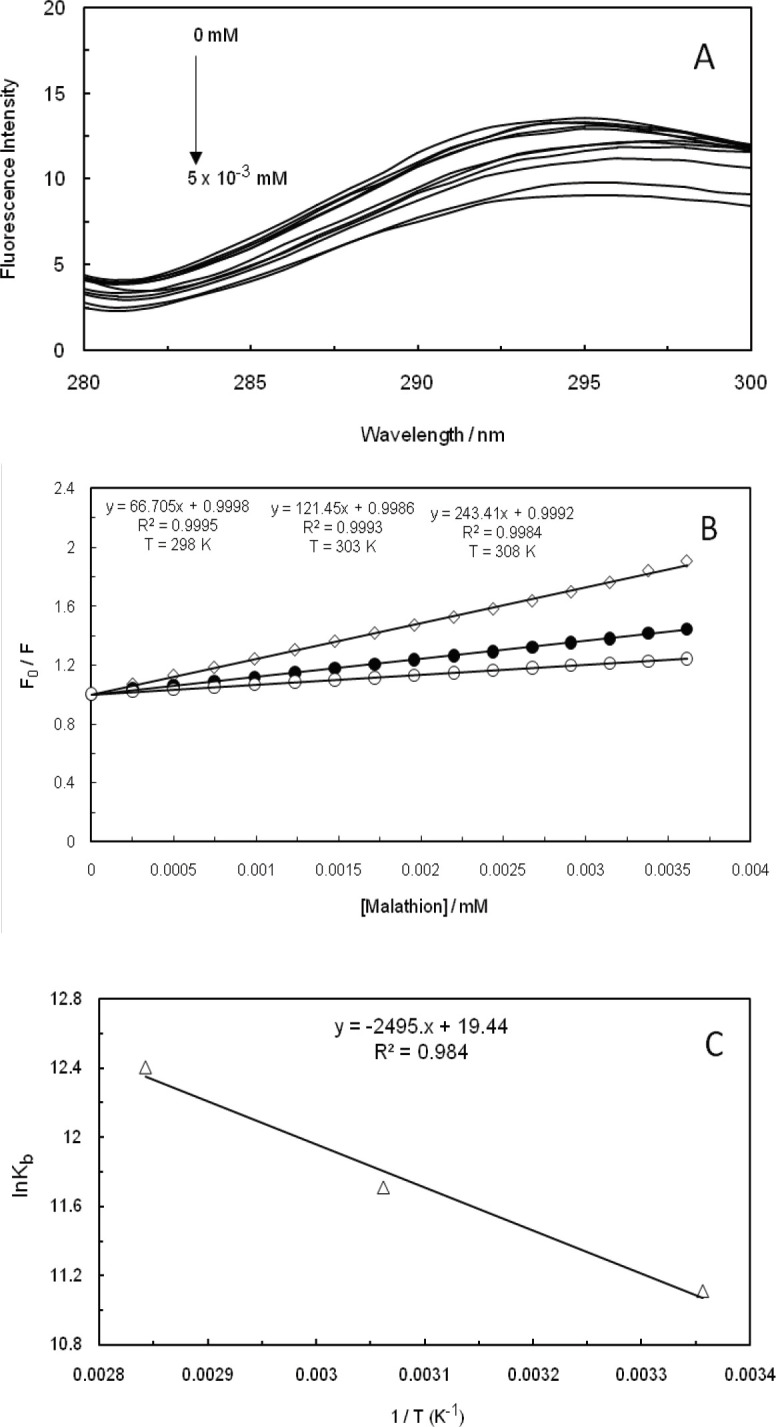
(A); Fluorescence spectra of ctDNA-malathion in the presence of a 1.2 GHz electromagnetic field at different concentrations (λex= 260 nm). (B); Stern-Volmer plots of fluorescence quenching of ctDNA-malathion in the presence of a 1.2 GHz electromagnetic field at different temperatures. (C); van't Hoff plot for the interaction of ctDNA-malathion in the presence of a 1.2 GHz electromagnetic field

Fluorescence quenching refers to any process that is capable of decreasing the fluorescence intensity of a sample. In fact, two quenching processes are known, including static and dynamic, and they both require the existence of molecular contact between the fluorophore and quencher. Static quenching refers to the formation of a non-fluorescent fluorophore-quencher complex. On the other hand, dynamic quenching stands for quencher diffusion to fluorophore during the lifetime of excited state, and upon contact, fluorophore returns to the ground state without the emission of a photon (36). The fluorescence quenching spectrum of malathion at the excitation wavelength of 260 nm, as the amounts of ctDNA had been increased in the absence and presence of EMF (100 kHz, 1.2 GHz), is displayed in Figures 3A, 4A, and 5A. The fluorescence intensity of ctDNA was detected to regularly decrease with the enhancement of malathion concentration in the absence and presence of EMF at low and high frequencies (100 kHz, 1.2 GHz), being indicative of malathion capability in interacting with ct-DNA. In dynamic quenching, the biomolecular quenching constant is anticipated to increase as the temperature is heightened.

The fluorescence quenching data were analyzed through the Stern-Volmer equation (37-41):

F_0_/F= 1+ K_SV_ [Q]                     (1) 

Where F0 and F stand for fluorescence intensities in the absence and presence of quencher, respectively, [Q] is the concentration of quencher, and K_SV_ represents the Stern-Volmer quenching constant. The plots of the Stern-Volmer equation at different temperatures (298 K, 303 K, and 308 K) are shown in Figures 3B, 4B, and 5B in the absence and presence of EMF at low and high frequencies (100 kHz, 1.2 GHz). The K_SV_ that had been obtained from this equation is presented in Table 1 and as it can be observed, the values were enhanced by heightening the temperature, suggesting that the mechanism of quenching were dynamic in the absence and presence of EMF (100 kHz, 1.2 GHz). Thermodynamic parameters in an interaction are considered as evidence for confirming the existence of binding forces. If the enthalpy change (∆H^0^) does not significantly vary over the temperature range that had been studied, then its value and that of entropy change (∆S^0^) can be determined from the van’t Hoff equation (28, 42-44):

log K = - ∆H^0^/ (2.303 RT) + ∆S^0^/ (2.303R)                      (2)

∆G^0^= ∆H^0^-T∆S^0^                      (3)

Where K is the binding constant at three specific temperatures (298 K, 303 K, and 308 K), R would be the gas constant, and T represents the absolute temperature. The ∆H^0^ and ∆S^0^ values were obtained from the slope and intercept of the linear van’t Hoff plot, which is based on lnK versus 1/T. We have evaluated the Gibbs free energy change (∆G^0^) from equation (3) (45-48), while the negative change of standard Gibbs free energy suggested that the mode of binding is apparently a spontaneous process. The values of ∆H^0^, ∆S^0^, and ∆G^0^ are listed in Table 1. It was indicated by the positive enthalpy and entropy values that the mode of binding was an endothermic and entropy-increasing process. It can be also suggested that hydrophobic force plays an essential role throughout the interaction between malathion and ctDNA in the absence and presence of EMF, which coincides with the other available results (Figures 3C, 4C, and 5C). 

***Thermal denaturation studies***

**Figure 6:**
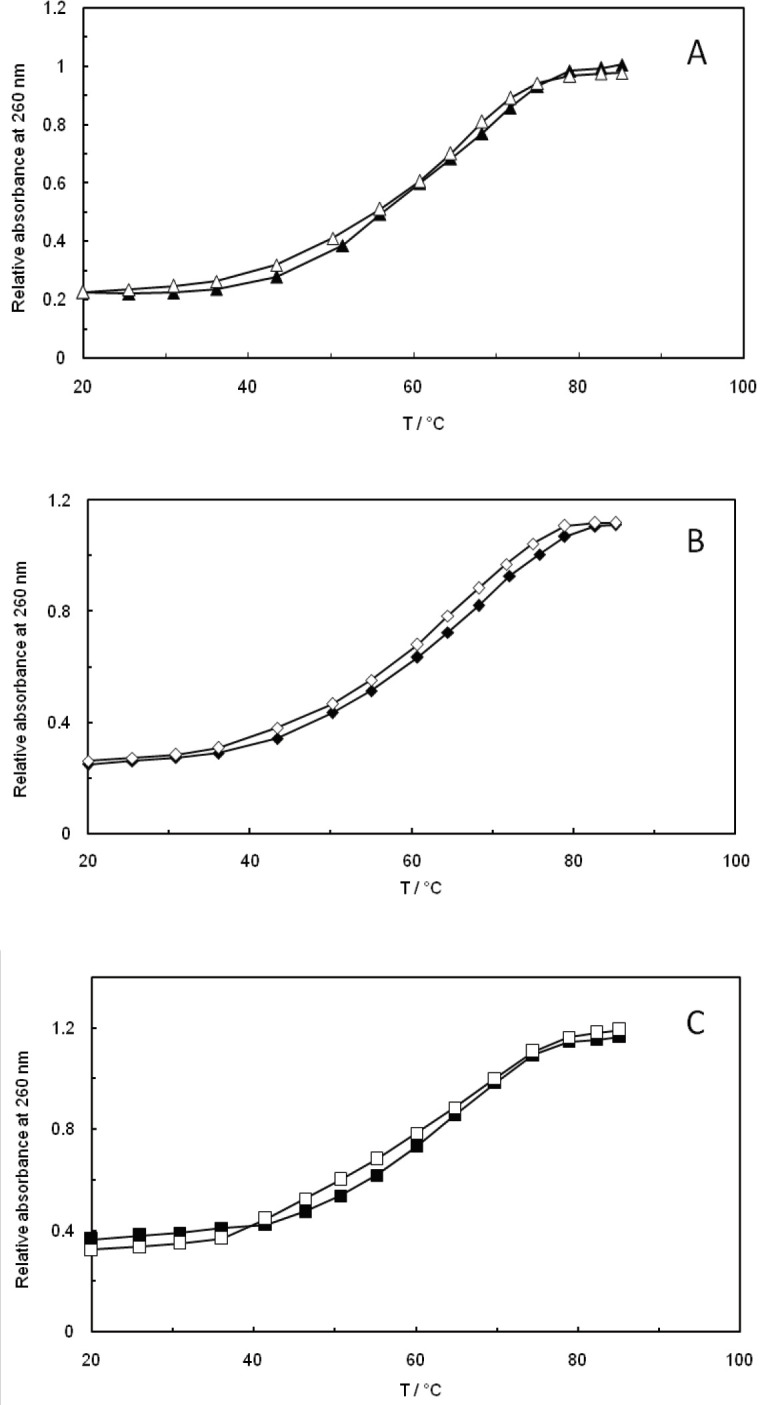
(A); Optimal thermal melting profiles of ctDNA-malathion (∆) ctDNA, (▲) ctDNA-malathion. (B); Optimal thermal melting profiles of ctDNA-malathion in the presence of 100 kHz electromagnetic field (◊) ctDNA, (♦) ctDNA-malathion. (C); Optimal thermal melting profiles of ctDNA-malathion in the presence of 1.2 GHz electromagnetic field (□) ctDNA, (■) ctDNA-malathion

The transition point (T_m_) of DNA is dependent on the strength and mode of its interaction with small molecules. In general, groove binding or electrostatic binding, along with the phosphate backbone of DNA, can only cause a small alteration in thermal denaturation temperature, while intercalation leads to a significant rise in the thermal denaturation temperature of DNA due to the stabilization of duplex temperature. Therefore, the thermal denaturation experiment of DNA can provide a convenient tool for detecting the designated binding, as well as assessing the relative strengths (49-52). Considering the ctDNA-malathion melting curves in the absence and presence of EMF (100 kHz, 1.2 GHz) that are illustrated in Figure 6 (A-C), it is notable that the detected change in T_m_ is very little, which supports the observations that had claimed the binding mode to be non-intercalative. The small increase in T_m_ was probably due to the induced conformational alterations of ctDNA that had been caused by the groove binding of malathion with ctDNA in the absence and presence of EMF at low and high frequencies (100 kHz, 1.2 GHz).

***Viscosity measurements***

**Figure 7:**
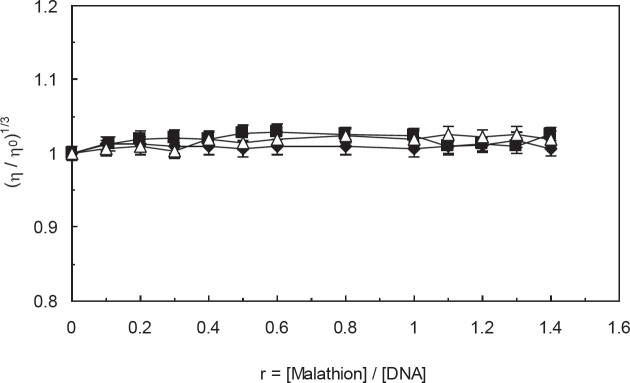
Effect of increasing amounts of malathion on the relative viscosity of ctDNA in the absence and presence of electromagnetic fields, (∆) r = [malathion ]/[ctDNA], (♦) r = [malathion ]/[ctDNA] in the presence of 100 KHz electromagnetic field, (■) r = [malathion ]/[ctDNA] in the presence of 1.2 GHz electromagnetic field

Viscosity measurement is sensitive towards the induced changes in the length of DNA and is regarded as the least ambiguous and critical test for determining the binding mode of a solution (53). A classical intercalation model is known to cause a significant increase in the viscosity of DNA solution that is due to increased length of the DNA helix. In contrast, the non-intercalation bindings, such as electrostatic and groove force mode, have not been detected to cause any obvious increase in DNA viscosity (54). We have procured a viscosity plot of (µ/µ_0_)^1/3^ versus [malathion ] / [ctDNA] to study the occurrence of any changes in the viscosity of ct-DNA solution throughout the absence and presence of EMF (100 kHz, 1.2 GHz). As can be seen in Figure 7, through the continuous addition of malathion to ctDNA solution in the absence and presence of EMF (100 kHz, 1.2 GHz), the amount of increase in viscosity had been so little that it has not been as pronounced as observed for classical intercalators. Consequently, this fact confirms that the manner of malathion interaction to ctDNA is in groove mode throughout the absence and presence of EMF (100 kHz, 1.2 GHz).

***Effect of ionic strength***

**Figure 8:**
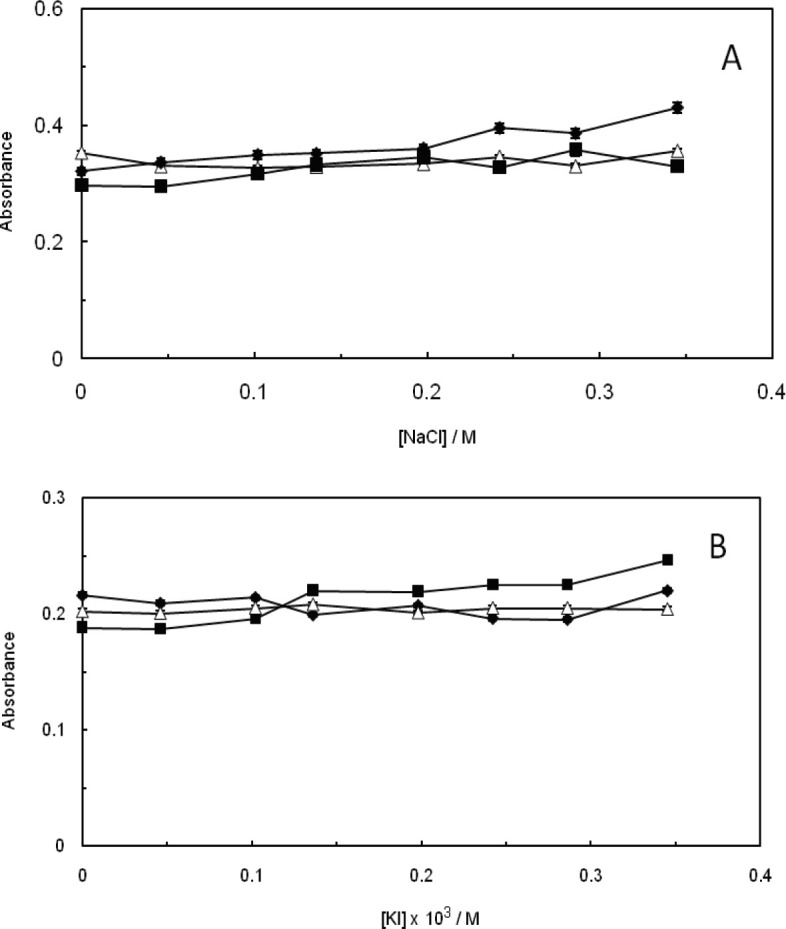
(A); Effect of ionic strength on the absorbance of ctDNA-malathion complex in the presence of NaCl, (∆)(ctDNA-malathion) NaCl, (♦) (ctDNA-malathion) NaCl in the presence of 100 kHz electromagnetic field, (■) (ctDNA-malathion) NaCl in the presence of 1.2GHz electromagnetic field. (B); Effect of ionic strength on the absorbance of ctDNA-malathion complex in the presence of KI, (∆) (ctDNA-malathion) KI, (♦) (ctDNA-malathion) KI in the presence of 100 kHz electromagnetic field, (■)(ctDNA-malathion) KI in the presence of 1.2 GHz electromagnetic field

The effect of ionic strength is an efficient method to recognize the existing binding mode between small molecules and DNA. The small molecules that bind strongly to DNA are usually composed of a charged component, however, if the electrostatic binding interaction contains a dominant role throughout the binding interaction of DNA with small molecules, then the strength of the interaction is supposed to decrease as the salt concentration is enhanced within the system (10, 55). Concerning groove binding, a small molecule binds in the groove of DNA duplex and is exposed to the surrounding much more than what occurs in an intercalation case (56). The experimental results have shown that the absorbance of the malathion -ctDNA complex, in the absence and presence of EMF (100 kHz, 1.2 GHz), has not faced any alteration as the NaCl and KI concentrations enhanced. Therefore, it can be suggested that the groove binding mode stands as the main interaction of malathion with ctDNA in the absence and presence of EMF (Figures 8A and 8B).

***Circular dichroism (CD) spectroscopy***

**Figure 9:**
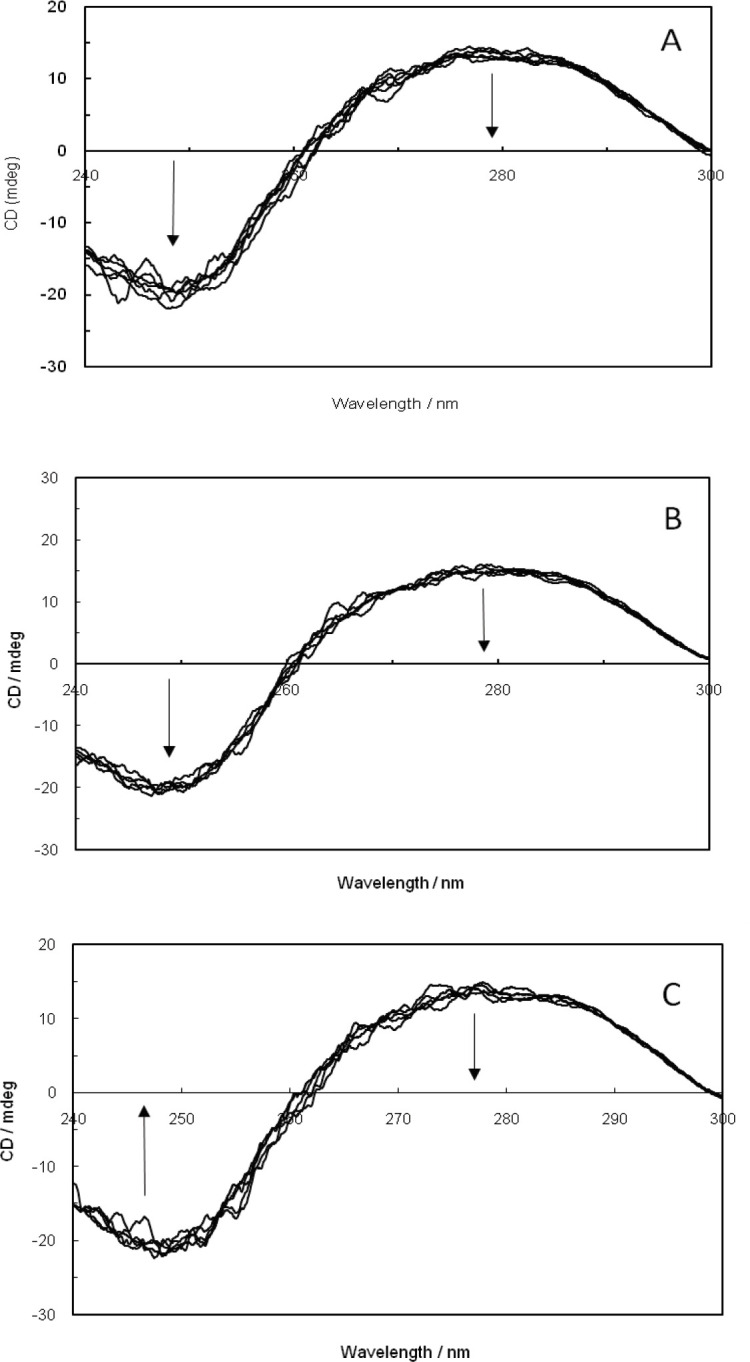
(A); Circular dichroism spectra of ctDNA in the presence of increasing amounts of malathion. (B); ctDNA-malathion in the presence of 100 kHz electromagnetic field. (C); ctDNA-malathion in the presence of a 1.2 GHz electromagnetic field

Due to being very sensitive toward induced changes in the secondary structure of bio-macromolecules, circular dichroism spectroscopy is employed to detect such alterations in DNA upon its interaction with small molecules (57, 58). Groove binding and electrostatic interaction of small molecules have displayed less or zero perturbation on the base stacking and helicity bands, whereas intercalation can significantly alter the intensities of both bands and consequently stabilize the right-handed B conformation of DNA (59). As illustrated in Figure 9 (A, B), the existing positive and negative bands in CD spectra have decreased and faced a remarkable increase in the ellipticity of ctDNA-malathion complex formation as the malathion concentration in ctDNA solution had been enhanced in the absence and presence of EMF (100 kHz). Thus, there may be a possibility that malathion binds to ctDNA through a groove binding mode. On the other hand, Figure 9C demonstrates the CD spectrum of the ctDNA-malathion complex in the presence of 1.2 GHz EMF. The observed decrease in positive and negative bands of ctDNA was likely caused by the occurrence of a transition from a more B-like to a more C-like structure, which is expressive of a non-intercalative interaction between malathion and ctDNA in the presence of 1.2 GHz EMF.

***Comparison of the interactions of malathion with ss ctDNA and ds ctDNA***

**Figure 10:**
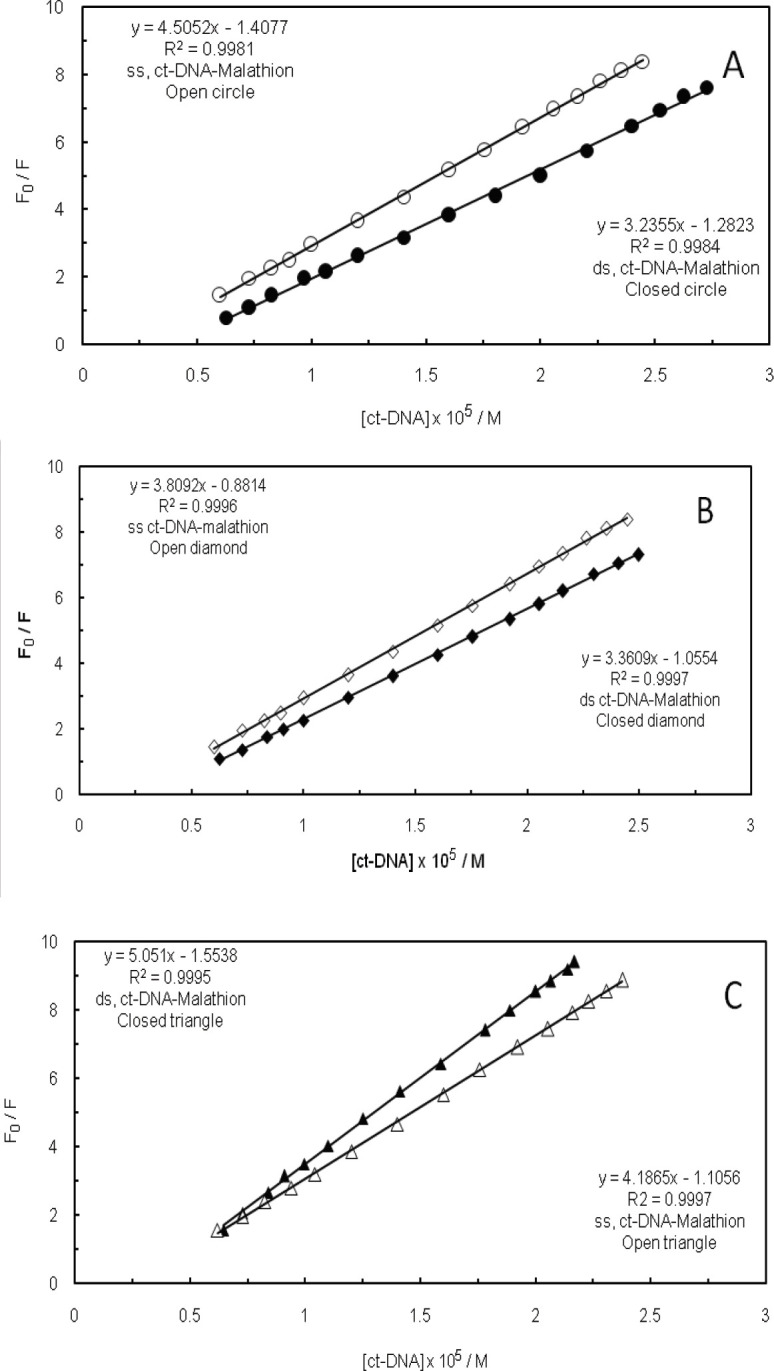
(A); Stern-Volmer plots of malathion by (○) ss ctDNA and (●) ds ctDNA. (B); Stern-Volmer plots of malathion in the presence of 100 kHz electromagnetic field by (◊) ss ctDNA and (♦) ds ctDNA. (C); Stern-Volmer plots of malathion in the presence of 1.2 GHz electromagnetic field by (∆) ss ctDNA and (▲) ds ctDNA

We have compared the behavior of native and denatured structures of DNA in the presence of malathion and EMF with low and high frequencies. For this purpose, double-strand DNA was converted into a single-strand DNA with the opening of its double helix, which had been achieved by incubating at 100 °C for 30 min, followed by rapid cooling in ice water (60). As it is shown in Figure 10 (A, B), the K_SV_ values of ctDNA-malathion complex (both states of ctDNA, ss ctDNA and ds ctDNA), in the absence and presence of 100 kHz EMF, indicated that malathion interacts with the base pairs of duplex ctDNA through a minor or major groove binding. As it can be observed in Figure 10C, in the presence of 1.2 GHz EMF, the K_SV_ value of ds ctDNA-malathion was higher than ss ctDNA-malathion, which is suggestive of an intercalator binding mode. Moreover, these K_SV_ values have exhibited that EMF had caused the different behaviors of ctDNA-malathion complex formation and have also displayed the essential functionality of EMF throughout formation of the ctDNA-malathion complex.

***Molecular modeling***

**Figure11:**
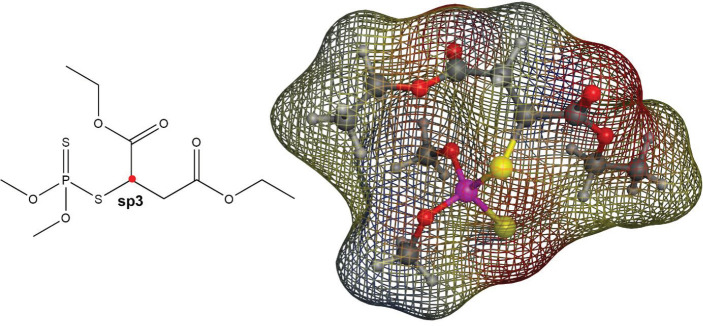
Malathion 2D and 3D structures. The color of the meshed surface map around malathion is based on the electrostatic forces around the atom types. (red for negative charges, blue for positive charges, and yellow for neutral)

**Figure12:**
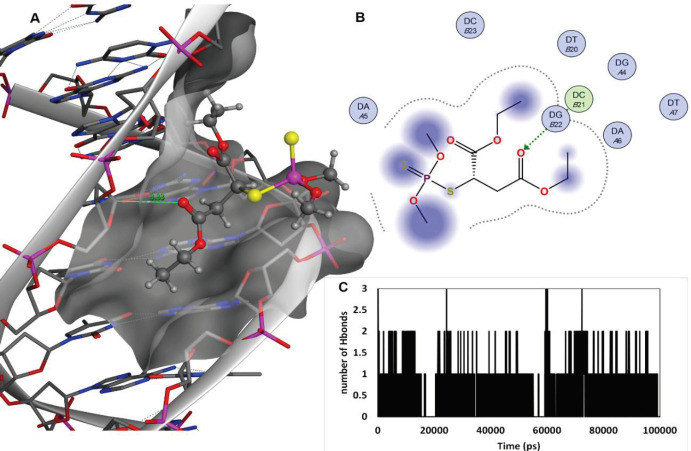
Best docking results of ctDNA-malathion interaction (Pose 1). (A); malathion in a groove binding site of B-DNA, the active site surface map is added, malathion is in ball and stick and B-DNA nucleotides are in sticks. The sole H bond is in green with a measured distance of 3.33 nm. (B); the 2D malathion interaction site. (C); the number of H bonds formed during the course of 100 ns MD simulation

**Figure 13:**
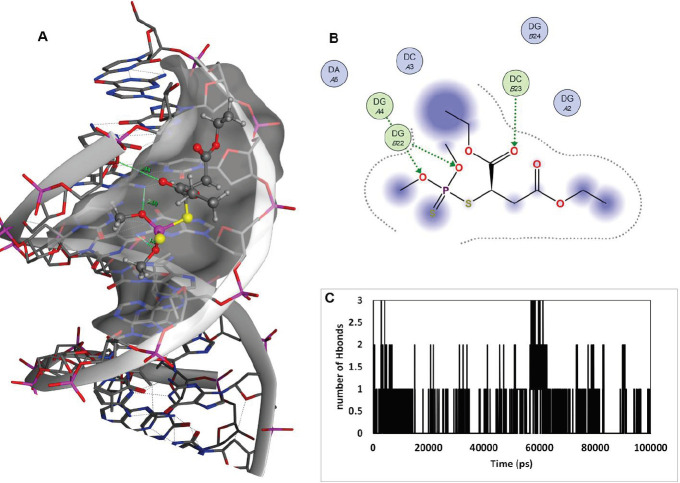
Second-best docking results of ctDNA-malathion interaction (Pose2). (A); malathion in a groove binding site of B-DNA, the active site surface map is added, malathion is in ball and stick and B-DNA nucleotides are in sticks. Three H bonds are in green with the measured distance of 3.44, 2.99, and 3.05 nm. (B); 2D malathion interaction site. (C); Number of H bonds formed during the course of 100 ns MD simulation

**Figure 14:**
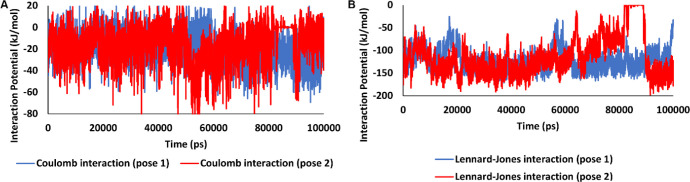
Interaction potentials between B-DNA and malathion (Pose 1 in blue and Pose 2 in red). (A); Coulomb interaction, (B); Lennard-Jones interaction

The structure of malathion contains a symmetry around the central carbon with sp3 hybrid (Figure 11) that can interact with protein active sites from the two ethyl acetate arms (symmetrically) or one ethyl acetate and one thiophosphate arm (asymmetrically). After repeating the docking study with free binding poses, malathion exhibited a strong affinity for binding to the B-DNA groove binding sites, which is indicative of its ability in binding to the B-DNA active sites in both conformations. The best docking pose of malathion -ctDNA interaction is shown in Figure 12, in which malathion is symmetrically linked to B-DNA (Figure 12A). Although one H bond stands as the only stabilizer of interaction, yet it does not face any alteration in the course of MD simulation. The second-best docking pose is demonstrated in Figure 13. In this conformation, malathion is asymmetrically bound while three H bonds are formed to stabilize the structure. However, a simulation study has not approved the complete stability of these H bonds and as displayed in Figure 13C, their interactions are very loose and had been lost in the last nanoseconds of MD simulation. The best docking scores and possible interactions, along with the energy of bindings, are summarized in Table 2. To investigate the non-bonded interaction potentials, we have taken advantage of the existing Lennard-Jones interactions between the two atoms and Coulomb interactions between the two charged particles. These interactions were analyzed in the course of the simulation studies and summarized in Figure 14. The measured coulomb and Lennard-Jones potentials for the first pose were -19 ± 3.3 and -122 ± 5.1, and for the second pose were observed to be -16 ± 2.6 and -114 ± 11, respectively. Besides the more potent interactions between malathion and B-DNA in the first pose, it can be understood from Figure 14 that these potentials have not been stable throughout the second pose. As it can be perceived, the changes have increased especially after 70 ns simulation, which might be due to the separation of the ligand from ctDNA base pairs. 

**Table 1:** Thermodynamic parameters of (ctDNA-malathion) system in the absence and presence of electromagnetic field (100 kHz, 1.2 GHz) at different temperatures, pH 6.8, (λex= 260 nm)

System	T / K	Ksv 10^-4^ M^-1^	Ka 10^-4^ M^-1^	G^0^ kJ.mol^-1^	H^0^ kJ.mol^-1^	S^0^ J.mol^-1^K^-1^
	298	6.18 0.12	5.37 0.12	-26.98		
ctDNA-malathion	303	13.70 0.12	12.42 0.12	-29.55	16.31	146.51
	308	16.67 0.12	15.19 0.12	-30.55		
	298	6.67 0.08	5.55 0.08	-27.06		
ctDNA-malathion (100 kHz)	303	12.14 0.08	10.64 0.08	-29.16	20.75	161.65
	308	24.34 0.08	23.05 0.08	-31.62		
	298	8.58 0.15	6.84 0.15	-27.58		
ctDNA-malathion (1.2 GHz)	303	13.61 0.15	12.38 0.15	-29.54	15.19	145.29
	308	22.73 0.15	20.19 0.15	-31.28		

**Table 2:** Results of the best docking poses between B-DNA and malathion, possible interactions, and binding energies

**Docking poses**	**Interactions **				**Binding energy (kcal/mol)**
	Malathion atoms	B-DNA atoms	Distances (nm)	Interaction Energy (kcal/mol)	
**Pose 1**	O19 (ethyl acetate)	C1 (DC)	3.33	-0.6	-6.15
**Pose 2**	O1 (ethyl acetate)	C1 (DC)	3.44	-1.2	-5.7
	O9 (thiophosphate)	N2 (DG)	2.99	-1.8	
	O11 (thiophosphate)	N2 (DG)	3.05	-1.9	

## Discussion

Small molecules can bind to DNA via covalent or non-covalent interactions, which results in altering or inhibiting the function of DNA. The most common type of interaction is intercalation or groove binding. Previous studies have shown that intercalation of the small molecules into DNA base pairs can lead to massive destruction of the DNA’s helicity and functionality. Thus, understanding the interaction patterns of medicines or other small molecules can be very crucial.

As said in the introduction section, malathion is an organophosphorus pesticide that can be found in agricultural products. The importance of the effects of this substance on the nucleic acids can be guidance for further pharmaceutical designs or even toxicology studies.

In this study, the interaction between malathion and calf thymus DNA (ctDNA), in the absence and presence of EMF at low and high frequencies, was investigated through various spectroscopies and viscosity measurements.

RLS studies of the ctDNA-malathion complex indicated that the interaction behavior of ctDNA-malathion in the presence of 1.2 GHz EMF was different from that of the ctDNA-malathion complex in the presence of 100 kHz EMF. The alterations of RLS intensity regarding ctDNA-malathion, in the presence of 1.2 GHz, have displayed and pointed out the more conformational changes of ctDNA upon interaction with malathion in the presence of 1.2 GHz than the case of 100 kHz. It was clearly revealed that EMF leaves its effects on ctDNA in high frequency with its complex formation behavior. Therefore, by observing the ∆IRLS values of ctDNA-malathion in the presence of 1.2 GHz EMF, it can be determined that ctDNA-malathion complex formation contains a different behavior in the presence of EMF with high frequency when compared to the case of low frequency. On the other hand, ctDNA and malathion are ionized in the presence of EMF, therefore, more ionizable groups in ctDNA and malathion can cause more binding affinity between them. EMF at high frequency (1.2 GHz) can induce more ionizable groups than at low frequency (100 kHz) in ctDNA and malathion, which shows the ctDNA-malathion complex formation at 1.2 GHz EMF behaves different than 100 kHz. 

The competitive interaction of AO and EB binding to ctDNA-malathion complex in the presence of EMF showed that malathion did not intercalate in the ctDNA base pairs; therefore, an organophosphorous compound does not induce the genetic aberrations in humans with utilizing of agricultural products.

Fluorescent measurements of the malathion-ctDNA complex suggested that malathion interacts with ctDNA through a noncovalent interaction in the groove binding mode. The interaction forces between small molecules and biomolecules include hydrogen bonds, hydrophobic force, van der Waals force, and electrostatic interactions. Dynamic quenching has clearly confirmed that the binding site of malathion on ctDNA, in the absence and presence of EMF (100 kHz, 1.2 GHz), was in the form of groove binding. Therefore, electromagnetic fields at low and high frequencies have not caused any changes in the binding site of malathion on ctDNA.

The viscosity measurements did not show significant changes in the ctDNA viscosity after addition of malathion. This can be a result of groove binding interaction rather than intercalation. On the other side, the increase in the salt concentrations reduced the strength of the interaction between ctDNA and malathion, which is due to the non-covalent electrostatic interactions.

CD spectroscopy results also confirm the theory of groove binding for malathion in ctDNA; the transition from B-form to the C-form of the ctDNA after the increase of the malathion concentration showed an overall instability in the whole structure at 1.2 GHz EMF.

Molecular docking and dynamics interaction studies of malathion and a sample B-DNA have shown that groove binding sites can be considered the most befitting active sites for malathion to bind. On the other hand, the orientation and conformation of malathion in an active site are very important in regard to the stability of interaction. The performed molecular dynamics studies have indicated that when malathion binds symmetrically to the groove binding site, the best interaction potentials can be observed and at least one stable H bond can be expected to form. Although we could not simulate the effects of electromagnetic forces in our studies, yet we were able to conclude that polarization of solvent, ctDNA, and ligand is effective throughout the binding poses of malathion in an active site.

## Conclusion

Most ligands tend to interact non-covalently with DNA through two general selective modes: a groove-bound fashion that is stabilized by a mixture of hydrophobic, electrostatic, and hydrogen bonding interactions and an intercalative association in which a planar, heteroaromatic moiety slides in between the DNA base pairs. The non-covalent binding of small molecules to DNA results in the occurrence of conformational and behavior alterations that can affect the application of DNA. In this work, the interaction of malathion with ctDNA, in the absence and presence of EMF at low and high frequencies, was studied through the means of different spectroscopic and viscosity methods that involved the usage of EB and AO as probes. The spectroscopic and melting results have indicated that the interaction between malathion and ctDNA in the absence and presence of EMF was not intercalation and probably groove binding. On the other hand, comparing the interaction between malathion with ss ctDNA and ds ctDNA in the presence of EMF with high frequencies has determined that in a condition with high frequency, EMF can change the binding site of malathion to ctDNA. Consequently, it is indicated that EMF plus high frequency can induce two sets of binding sites of malathion to ctDNA in a simultaneous manner. This research can help to clarify the molecular mechanism of malathion in vivo and provide a guide for its usage doses in the field of agriculture. 

## Authots' Contributions

JC, MRS, and ZAT designed the research study. TS, MAL, ZS, and ZAT performed the experiments and collected the data. TS, ZAT, and JC analyzed the data. TS, MAL, ZS, and ZAT wrote the initial manuscript. MRS and JC revised the manuscript. All authors discussed the results and contributed to the final manuscript. 

## Conflicts of Interest

 The authors declare no competing interests.
